# Supplementary material for: Emotion Recognition in Preterm and Full-Term School-Age Children
Source: Int J Environ Res Public Health. 2022 May 26;19(11):6507. doi: 10.3390/ijerph19116507 (PMC9180201; doi:10.3390/ijerph19116507)
Supplement: Supplementary file 1 [file ijerph-19-06507-s001.zip › ijerph-1706627-supplementary.pdf]

SUPPLEMENTARY MATERIALS

ID	GA	BW	Apgar 1min	Apgar 5min	Apgar 10min	Cranial Circ.	Lenght	Mecanical Ventialtion	Parentelar Nutrition	NICU stay	Difficulties at birth	Start wolking	First words	NDDs
1	31+3	1550	9	9	-	28,5	40	No	Yes (5 days)	Yes (22 days)		12 months	18 months	
2	30+3	1480	4	8	-	28,5	42	Yes (4 days)	Yes (7 days)	Yes (36 days)	bilateral periventricular hypercogenicity, then normalized	8 months	12 months	Dyslexia
3	30+3	1632	8	9	-	29	43	Yes(7 days)	Yes (7 days)	Yes (36 days)		9 months	12 months	
4	28+0	745	8	9	9	24,5		Yes(68 days)	Yes (42 days)	Yes	hypotension (support with inotropic drugs for for 11 days)			
5	27+4	862	1	3	5	24,5	36	Yes (77 days)	Yes (33 days)	Yes (81 days)	intubation at birth	16 months	18 months	
6	26+3	800										16 months	12 months	
7	28+0	1230												
8	28+0	1500												
9	26+5	896	5	7	8	25,2	30	Yes (32 days)	Yes (42 days)	Yes	intubation at birth for 1 month, ipotension (dopamine drug), antireflux theraphy, retinopathy stage 3 (laser operation and resuolution within a month)	12 months	12 months	
10	28+2	850	7	7	8	25,4	33,5	Yes (18 hours)	Yes (16 days)	Yes (56 days)	intubation, but then normal respiration	18 months	12 months	
11	31+4	1285	6	8	9	28	39	Yes (2 days)	Yes (19 days)	Yes				
12	31+4	1950	4	6	7	29,5	46	Yes (7 days)	Yes (20 days)	Yes				
13	29+2	1675	8	8	9	27,7	42	Yes (1 day)	Yes (17 days)	Yes (40 days)	moderate perivntricular hyperechogenicity in the frontal and occipital areas, diffuse microgranule	17 months	12 months	
14	31+4	1345	8	8	-	29	39	Yes (8 hours)	Yes (12 days)	Yes (27 days)	mild perivntricular hyperechogenicity, foramen ovale pervio	18 months	24 months	
15	32+5	1985	7	9	-	31,1	41	Yes (1 day)	Yes (5 days)		oxygen teraphy (1 day), discoagulopathy (plasma transfusion), doubtful cochlear function	15 months	26 months	
16	31+1	1820	7	7	9	31,3	42	Yes (1 day)	Yes (only for idratation)	Yes (28 days)	apnea of prematurity (caffeine drug for 2 weeks)	9 months	11 months	
17	27+5	880	8	9	9	26,2	36	Yes (17 days)	Yes (42 days)	Yes	slight right periventricular hyperechogenicity, slight ventricular asymmetry (left> right),mild bilateral pulmonary hypotransparency	13 months	12 months	
18	34+0	2270						Yes	Yes (42 days)	Yes		13 months	12 months	
19	30+0	2100										16 months	11 months	borderline for ADHD
20	28+0	990	9	9	-	25	35	Yes (19 days)	Yes (13 days)	Yes (25 days)	mild periventricular hyperechogenicity in fronto-parietal and occipital areas, hyperbilirubinemia (resolved with phototherapy)			
21	28+0	1100	6	7	8	26	36	Yes (24 days)	Yes (11 days)	Yes		18 months	12 months	
22	24+0	656	8	8	-	22	30	Yes (21 days)		Yes	periventricular hyperechogenicity, ledt pneumothorax, coffeine for apnea	17 months	36 months	Dysgraphia
23	30+0	950	9	10	-	26	34,5	No		Yes (40 days)	mild periventricular hyperechogenicity in occipital part of cerebellum, apnea of prematurity	17 months	24 months	
24	25+3	640	7	8	9	22,9	33	Yes	Yes (14 days)	Yes	apnea of prematurity,	18	12	

											foramen ovale pervio, bilateral retinopathy stage 2	months	months	
25	29+0	1350								Yes		10 months	12 months	
26	32+2	1950	9	10	-	29	43	Yes (6 days)	Yes (7 days)	Yes		14 months	12 months	
27	27+6	512	7	8	-	24	35	Yes (18 days)	Yes (28 days)	Yes	pneumothorax	18 months	12 months	
28	34+0	1145	9	9	-	29,5	39	No	Yes (9 days)	Yes		19 months	6 months	
29	36+0	2300										13 months	7 months	
30	30+0	900												
31	30+0	1200												
32	32+3	1980	9	9	9			No	Yes (3 days)			15 months	14 months	
33	32+3	2200	7	9	9			No	Yes (1 day)			17 months	15 months	
34	32+5	2500	8	8	9	32,6	48	Yes (4 days)	Yes (5 days)	Yes (18 days)		13 months	24 months	

Table S1. Summary of neonatal information of preterm children. GA= gestational age; BW= birth weight; APGAR score at 1, 5 and 10 minutes after birth; Cranical circumference, Length, Days of mechanical ventilations; Days of parentelar nutrition; length of period spent in neonatal intensive care unit (NICU); Difficulties at birth, including neurological, respiratory, cardiac, gastrointestinal and sensory problems; age when the child moved the first steps and said the first words; NDDs= diagnosis for neurodevelopmental disorders

	**Full-term children**	**Preterm children**	**Test for group differences**
**CPM**<br>Z scores	0.86 (0.67)	0.77 (0.66)<br>LP: 1.03 (0.73)<br>VP: 0.76 (0.68)<br>EP: 0.48 (0.48)	t= 0.50 *p*= .619
**Digit span forward**<br>Z scores	0.06 (0.97)	-0.16 (0.81)<br>LP: -0.19 (0.98)<br>VP: -0.23 (0.86)<br>EP: 0.08 (0.49)	t= 0.85 *p*= .400
**Digit span backwards**<br>Z scores	0.66 (0.94)	0.34 (0.79)<br>LP: 0.27 (0.93)<br>VP: 0.39 (0.70)<br>EP: 0.26 (0.97)	t= 1.34 *p*= .190
**Attention Network Task** (ANT)	Alerting: 29.72 (41.04)<br><br><br><br>Orienting: 20.34 (63.84)<br><br><br><br>Control: 34.50 (51.84)	Alerting: 38.11 (50.62)<br>LP: 11.15 (58.55)<br>VP: 47.91 (45.97)<br>EP: 38.88 (20.88)<br>Orienting: 26.25 (39.23)<br>LP: 68.27 (43.84)<br>VP: 16.61 (47.10)<br>EP: 10.40 (42.80)<br>Control: 47.01 (61.96)<br>LP: 50.64 (46.23)<br>VP: 54.14 (65.08)<br>EP: 24.02 (50.80)	Alerting: t= -0.67 *p*= .505<br><br><br><br>Orienting: t= -0.35 *p*= .730<br><br><br>Control: t= -0.82 *p*= .417
**Berg Card Sorting Test** (BCST)	Errors: 27.02% (8.35)<br><br><br><br>Perseverative Errors: 14.25% (6.14)	Errors: 41.89% (10.81)<br>LP: 41.57 (10.99)<br>VP: 42.79 (10.72)<br>EP: 44.50 (8.35)<br>Perseverative Errors: 21.85% (7.94)<br>LP: 19.31% (6.52)<br>VP: 22.29% (9.24)<br>EP: 23.22% (11.14)	Errors: t= -5.67, *p*< .001<br><br><br><br>Perseverative Errors: t= -3.71, *p*< .001

	Non Perseverative Errors:<br>12.77% (7.44)	Non Perseverative Errors:<br>18.61% (5.68)<br>LP: 14.73% (5.55)<br>VP: 17.76% (10.76)<br>EP: 24.78% (19.5)	Non Perseverative Errors:<br>t= -2.08, *p*= .042

Table S2 Cognitive Tests: Descriptive statistics divided for level of prematurity

	**Full-term children**	**Preterm children**	**Test for group differences**
**SDQ**	Difficulties Score: 5.00 (3.2)<br>Prosocial Behavior: 8.65 (1.4)<br>Emotional Symptoms: 1.24 (1.1)<br>Conduct Problems: 1.06 (1.0)<br>Hyperactivity: 2.12 (1.8)<br>Peer Problems: 0.59 (1.1)	Difficulties Score: 8.03 (4.3)<br>LP: 5.83 (2.14)<br>VP: 8.33 (3.90)<br>EP: 9.14 (6.31)<br>Prosocial Behavior: 8.10 (1.5)<br>LP: 8.33 (1.51)<br>VP: 8.05 (1.55)<br>EP: 8.00 (1.73)<br>Emotional Symptoms: 2.19 (1.9)<br>LP: 1.33 (1.21)<br>VP: 2.22 (1.63)<br>EP: 2.86 (2.97)<br>Conduct Problems: 1.61 (1.2)<br>LP: 1.17 (1.60)<br>VP: 1.67 (1.03)<br>EP: 1.85 (1.34)<br>Hyperactivity: 3.16 (2.0)<br>LP: 2.50 (1.38)<br>VP: 3.33 (1.97)<br>EP: 3.29 (2.56)<br>Peer Problems: 1.06 (1.3)<br>LP: 0.83 (1.17)<br>VP: 1.11 (1.32)<br>EP: 1.14 (1.46)	Difficulties Score: t= -2.75 *p*= .009*<br>Prosocial Behavior: t= 1.25 *p*= .219<br>Emotional Symptoms: t= -2.15 *p*= .037*<br>Conduct Problems: t= -1.74 *p*= .090<br>Hyperactivity: t= -1.87 *p*= .069<br>Peer Problems: t= -1.33 *p*= .191
**ERC**	Emotional Negativity: 25.53 (4.7)<br>Emotional Regulation: 27.83 (2.9)	Emotional Negativity: 27.11 (3.5)<br>LP: 26.33 (2.16)<br>VP: 27.47 (3.14)<br>EP: 27.00 (5.13)<br>Emotional Regulation: 27.10 (3.3)<br>LP: 28.67 (3.05)<br>VP: 26.65 (3.50)<br>EP: 26.86 (2.27)	Emotional Negativity: t= -1.21 *p*= .238<br>Emotional Regulation: t= 0.80 *p*= .426
**TMCQ**	Surgency: 3.40 (0.5)<br>Effortful Control: 3.28 (0.9)<br>Negative Affect: 2.42 (0.5)	Surgency: 3.26 (0.40)<br>LP: 3.33 (0.48)<br>VP: 3.29 (0.49)<br>EP: 3.16 (0.28)<br>Effortful Control: 3.41 (0.5)<br>LP: 3.49 (0.28)<br>VP: 3.38 (0.61)<br>EP: 3.41 (0.29)<br>Negative Affect: 2.76 (0.4)<br>LP: 2.71 (0.44)<br>VP: 2.78 (10.76)<br>EP: 2.83 (0.56)	Surgency: t= 0.89 *p*= .384<br>Effortful Control: t= -0.55 *p*= .592<br>Negative Affect: t= -2.43 *p*= .022*
**BRIEF**	Total: 47.89 (7.0)<br>Behavioral: 50.67 (7.0)<br>Emotional: 48.94 (6.6)<br>Cognitive: 47.00 (8.0)	Total: 53.75 (8.3)<br>LP: 48.17 (6.27)<br>VP: 54.94 (8.77)<br>EP: 55.43 (7.25)<br>Behavioral: 52.19 (8.3)<br>LP: 48.83 (4.31)<br>VP: 52.17 (8.47)<br>EP: 55.14 (10.07)<br>Emotional: 51.77 (9.6)	Total: t= -2.64 *p*= .012*<br>Behavioral: t= -0.69 *p*= .496<br>Emotional: t= -1.21 *p*= .231<br>Cognitive: t= -2.99 *p*= .005*

		LP: 48.33 (5.09)<br>VP: 52.56 (10.29)<br>EP: 52.71 (11.24)<br>Cognitive: 54.71 (9.8)<br>LP: 48.67 (7.89)<br>VP: 56.28 (10.94)<br>EP: 55.86 (6.69)	
**PSI**	Total stress: 57.83 (12.7)<br>Parental distress: 20.44 (6.3)<br>Dysfunctional interaction: 18.39 (3.5)<br>Difficult Child: 19.00 (4.8)<br>Defensiveness Response: 12.56 (4.2)	Total stress: 67.30 (10.6)<br>LP: 67.50 (6.83)<br>VP: 65.53 (11.45)<br>EP: 71.43 (11.16)<br>Parental distress: 22.53 (4.5)<br>LP: 21.83 (2.86)<br>VP: 22.06 (4.78)<br>EP: 24.29 (4.96)<br>Dysfunctional interaction: 21.57 (4.8)<br>LP: 22.50 (4.18)<br>VP: 20.47 (5.33)<br>EP: 23.43 (3.31)<br>Difficult Child: 23.20 (5.4)<br>LP: 23.17 (1.94)<br>VP: 23.00 (6.11)<br>EP: 23.71 (6.07)<br>Defensiveness Response: 14.10 (3.6)<br>LP: 13.67 (2.50)<br>VP: 13.65 (3.97)<br>EP: 15.57 (3.60)	Total stress: t= -2.66, *p*= .012*<br>Parental distress: t= -1.24, *p*= .226<br>Dysfunctional interaction: t= -2.66, *p*= .011*<br>Difficult Child: t= -2.82, *p*= .008*<br>Defensiveness Response t= -1.31, *p*= .201

Table S3 Parent-reported questionnaires: Descriptive statistics divided for level of prematurity

	Full-term children	Preterm children
**ERT Tot**	73.55% (5.10)	69.80% (7.41)<br>LP: 70.09 (5.12)<br>VP: 70.21 (8.97)<br>EP: 68.37 (5.23)
**ERT Positive Emotions**	86.61% (9.12)	80.15% (12.35)<br>LP: 83.59 (14.15)<br>VP: 78.29 (12.56)<br>EP: 81.25 (10.21)
**ERT Negative Emotions**	61.46% (8.35)	58.82% (10.67)<br>LP: 59.38 (6.89)<br>VP: 59.70 (12.67)<br>EP: 55.80 (8.73)

Table S4 Percentage of correct responses at the Emotion Recognition Task (ERT): Descriptive statistics divided for level of prematurity
